# A central role for P2X7 receptors in human microglia

**DOI:** 10.1186/s12974-018-1353-8

**Published:** 2018-11-21

**Authors:** Laura Janks, Cristian V. R. Sharma, Terrance M. Egan

**Affiliations:** 1 Department of Pharmacology and Physiology, Saint Louis University School of Medicine, 1402 South Grand Boulevard, St. Louis, MO 63104 USA; 2 Celprogen Inc., 3914 Del Amo Blvd, Suite 901, Torrance, CA 90503 USA

**Keywords:** Ligand-gated ion channel, Purinergic receptor, Permeabilization, Dye uptake, Inflammation, Innate immunity, IL-1β, Inflammasome

## Abstract

**Background:**

The ATP-gated ionotropic P2X7 receptor (P2X7R) has the unusual ability to function as a small cation channel and a trigger for permeabilization of plasmalemmal membranes. In murine microglia, P2X7R-mediated permeabilization is fundamental to microglial activation, proliferation, and IL-1β release. However, the role of the P2X7R in primary adult human microglia is poorly understood.

**Methods:**

We used patch-clamp electrophysiology to record ATP-gated current in cultured primary human microglia; confocal microscopy to measure membrane blebbing; fluorescence microscopy to demonstrate membrane permeabilization, caspase-1 activation, phosphatidylserine translocation, and phagocytosis; and kit-based assays to measure cytokine levels.

**Results:**

We found that ATP-gated inward currents facilitated with repetitive applications of ATP as expected for current through P2X7Rs and that P2X7R antagonists inhibited these currents. P2X7R antagonists also prevented the ATP-induced uptake of large cationic fluorescent dyes whereas drugs that target pannexin-1 channels had no effect. In contrast, ATP did not induce uptake of anionic dyes. The uptake of cationic dyes was blocked by drugs that target Cl^−^ channels. Finally, we found that ATP activates caspase-1 and inhibits phagocytosis, and these effects are blocked by both P2X7R and Cl^−^ channel antagonists.

**Conclusions:**

Our results demonstrate that primary human microglia in culture express functional P2X7Rs that stimulate both ATP-gated cationic currents and uptake of large molecular weight cationic dyes. Importantly, our data demonstrate that hypotheses drawn from work on murine immune cells accurately predict the essential role of P2X7Rs in a number of human innate immune functions such as phagocytosis and caspase-1 activation. Therefore, the P2X7R represents an attractive target for therapeutic intervention in human neuroinflammatory disorders.

**Electronic supplementary material:**

The online version of this article (10.1186/s12974-018-1353-8) contains supplementary material, which is available to authorized users.

## Background

Microglia are the resident immune cells of the central nervous system (CNS). They are essential for maintaining homeostasis in healthy tissues [1] and impact development by sculpting postnatal neural circuits [2, 3]. Microglia also contribute to the neuroinflammation that accompanies a number of CNS pathologies including Alzheimer’s and Parkinson’s diseases [2, 4–6]. They do so by producing pro-inflammatory cytokines that stimulate self-proliferation and recruit peripheral immune cells in an attempt to clear the insult.

Immune cells sense tissue injury by recognizing damage-associated molecular patterns (DAMPs), one of which is adenosine triphosphate (ATP). The extracellular concentration of ATP ([ATP]_o_) is low in healthy tissue [7, 8] but rises at sites of stress and cellular injury, leading to activation of ionotropic P2X7 receptors (P2X7Rs) on immune cell membranes [9]. Activation of the P2X7R initiates innate immunity by promoting assembly of the caspase-1-activating platform known as the NLRP3 inflammasome [10–14]. The result is increased production and non-canonical release of pro-inflammatory cytokines (predominately IL-1β and IL-18) by mononuclear phagocytes [15], leading to greater inflammation and/or cell death [16] in a variety of species, including humans [17]. In the CNS, ATP acting on P2X7Rs promotes activation and proliferation of microglia while decreasing their phagocytic capacity [18–21] and triggers neurotoxicity by stimulating production of TNF-α, COX-2, IL-6, MMP-9, and reactive oxygen species [22–25].

In light of the role of P2XRs in murine CNS pathophysiology, a crucial next step is to investigate the involvement of these ion channels in humans. Therefore, the aim of the present study was to test the capacity of ATP acting through P2XRs to initiate or modulate the immune response of cultured primary human microglia. Human microglia express mRNA for both P2X4Rs and P2X7Rs [26]. We found that primary adult human microglia kept in culture express functional P2X7Rs but not P2X4Rs and that P2X7Rs modulate key components of innate immunity. We demonstrate that ATP-driven permeabilization of human microglia is selective for cations and requires the participation of one or more proteins downstream of the P2X7R, one of which may be a Cl^−^ channel. Moreover, our data strongly suggest that the portal responsible for the transport of large cations across the surface membrane of human microglia is not the channel pore of the P2X7R. Finally, we show that ATP limits the ability of cultured human microglia to phagocytose bacterial debris, suggesting that P2X7Rs regulate essential innate immune functions in the human CNS.

## Methods

### Materials

Dulbecco’s modified Eagle’s medium (DMEM), RPMI 1640, fetal bovine serum (FBS), penicillin, and streptomycin were purchased from Gibco by Life Technologies (Waltham, MA, USA). ATP, BzATP, EDTA, ethidium bromide, lipopolysaccharides from *E. coli* O55:B5 (LPS; L2880), dimethyl sulfoxide (DMSO), Lucifer yellow dilithium salt, 5(6)-carboxyfluorescein, carbenoxolone, probenecid, tannic acid, 4,4′-diisothiocyano-2,2′-stilbenedisulfonic acid (DIDS), and nigericin were purchased from Millipore-Sigma (St. Louis, MO, USA). BAPTA-AM, YO-PRO-1, YOYO-1, Fluo-4-AM, pHrodo Red *E. coli* BioParticles Conjugate, and pHrodo Green AM were purchased from Invitrogen/ThermoFisher (Carlsbad, CA, USA). A438079, A804598, BX430, and ^10^Panx inhibitory peptide were purchased from Tocris (Minneapolis, MN, USA). Complete growth differentiated media with serum (E37089-01-S), extracellular matrix-coated T25 or T75 culture flasks (E37089-01-T25,T75), and Xeno-free cell dissociation media (M37001-02CM) were obtained from Celprogen (Torrance, CA, USA).

### Cell culture

Frozen ampules of healthy male (Caucasian, 29 years old) and female (Caucasian, 30 years old) human microglia isolated from the CNS (cortex) were purchased from Celprogen Inc. Freshly thawed microglia were washed once in complete growth differentiated media with serum and spun down before being maintained and sub-cultured every 48 to 72 h on human extracellular matrix-coated T25 and T75 flasks (Celprogen) at 37 °C with 5% CO_2_ in a humidified atmosphere. The mouse macrophage cell line J774A.1 was obtained from ATCC (Manassas, VA, USA) and cultured in DMEM containing 10% FBS, 2 mM glutamine, 50 U/ml penicillin, and 50 μg/ml streptomycin. Human monocytic THP-1 cells from ATCC were grown in RPMI 1640 culture medium containing 10% FBS and supplemented with 0.05 mM ß-mercaptoethanol. HEK-293T cells from ATCC were maintained in DMEM containing 10% FBS, 2 mM glutamine, 50 U/ml penicillin, and 50 μg/ml streptomycin. HEK-293T cells were co-transfected with human P2X7R and fluorescent reporter plasmids using Effectene (Qiagen, Germantown, MD, USA).

### Gene expression

Microglia were disassociated from the culture flask using a xeno-free cell disassociation media after the fourth passage and were lysed by addition of 0.75 mL of Ribozol for total RNA extraction using Epoch Life Science RNA Spin Columns (Sugar Land, TX, USA). cDNA was subsequently synthesized using Bioline SensiFast cDNA synthesis kit (Meridian Life Science, Memphis, TN, USA). Real-time qPCR was then performed on the microglia cDNA to check for the presence of various genes associated with different microglial activation profiles utilizing the BioRad C1000 Thermal Cycler CFX96 Real-Time System (Hercules, CA, USA). All gene primer sets were run as two technical replicates with the use of the SYBR Green fluorophore (ThermoFisher). Cq values were averaged and standardized to GAPDH expression of the sample and then plotted to allow the comparison of each gene’s expression in culture.

To identify P2X7R single nucleotide polymorphisms, genomic DNA was extracted from the human microglia cells. Whole-exosome sequencing was performed by the NovoGene Corp (Chula Vista, CA). A total of 1.0 μg genomic DNA per sample was used as input material for the DNA library preparation, and sequencing libraries were generated using SureSelect Human All Exon kit (Agilent Technologies, CA, USA) following the manufacturer’s recommendations, and index codes were added to each sample. Captured libraries were enriched in a PCR reaction to add index tags to prepare for hybridization. Products were purified using AMPure XP system (Beckman Coulter, Beverly, USA) and quantified using the Agilent high-sensitivity DNA assay on the Agilent Bioanalyzer 2100 system.

### Patch clamp electrophysiology

We studied both attached and detached microglia. Attached microglia were grown on 13-mm collagen-coated glass coverslips, and free-floating microglia were scraped from 35-mm plastic tissue culture dishes. In both cases, cells were studied in a recording chamber positioned on the stage of a Nikon inverted microscope and continuously perfused with an extracellular solution (ECS) containing the following (in mM): 140 NaCl, 5.4 KCl, 2 CaCl_2_, 33 glucose, and 10 HEPES at pH 7.4. Whole-cell currents were recorded at room temperature with low resistance (2–4 MΩ), lightly fire polished, borosilicate glass electrodes (1B150F, World Precision Instruments, Sarasota, FL), and an Axopatch 200B amplifier (Molecular Devices, San Jose, CA) filled with a solution containing the following (in mM): 155 NaCl, 10 HEPES, and 10 EGTA at pH 7.4. The holding potential was − 60 mV except where noted otherwise. Data were filtered at 5 kHz during acquisition and digitized at 10 kHz using ITC-16 data acquisition hardware (Heka Electronics, Holliston, MA). Drugs were applied using triple-barreled theta glass and a Perfusion Fast-Step SF-77 System (Warner Instruments, Hamden, CT). Current-voltage curves were generated either by measuring peak agonist-gated currents (3 s) at a range of steady holding potentials or by measuring the current caused by a 500-ms ramp of voltage from − 90 to 30 mV.

In experiments studying currents after phagocytosis, microglia were grown on collagen-coated coverslips and incubated with 20 μg/mL pHrodo Red *E. coli* BioParticles Conjugate in ECS for 16–24 h prior to recordings. In experiments where microglia were pretreated with LPS, 1 μg/mL LPS was added to cells for 12–24 h prior to recordings. Data were analyzed offline using IGOR Pro (Wavemetrics, Tigard, OR) and GraphPad Prism 7 (La Jolla, CA, USA) softwares.

### Pharmacology

Repetitive applications of ATP and BzATP produce progressive facilitation of P2X7R-mediated currents [27, 28]. To avoid the confounding effect of facilitation on interpretation of concentration-response curves, we applied agonists no more than twice to single cells. We measured the peak current density (pA/pF) for 3-s applications of multiple concentrations of ATP and BzATP, and then we pooled the respective results to yield an average current density for each concentration. These data were plotted as log(agonist concentration) versus current density using Prism 7 (GraphPad, La Jolla, CA) and fit by nonlinear regression to calculate the concentration of agonist giving a half-maximal response (i.e., the EC_50_).

### Intracellular [Ca^2+^]

Human microglia, grown overnight on 13-mm collagen-coated glass coverslips, were incubated for 30 min in normal extracellular solution (+/− Ca^2+^) containing 5 μM Fluo-4-AM and 0.02% (*w*/*v*) Pluronic F-127 at room temperature, washed free of the reagents, and left for 30 min at 37 °C to permit de-esterification. Then, single coverslips were transferred to the 14-mm microwell of a MatTek Co (Ashland, MA) glass bottom culture dish positioned on the stage of an Olympus IX70 inverted microscope and visualized (excitation 494 nm, emission 514 nm) using a × 20 objective (0.75 N.A.). Images were captured at a rate of five frames per second using MicroManager [29]. Each image contained 30–50 cells defining regions of interest, and each experiment was repeated at least ten times. Data traces show the fold change in fluorescence over baseline after background subtraction (*F*/*F*_0_).

### Phagocytosis and caspase-1

Human microglia were grown in 35-mm dishes for 24–72 h. The cells were incubated with or without the P2X7R antagonist A438079 (50 μM) in normal extracellular solution for 1 h at 37 °C. To measure phagocytosis, the microglia were subsequently incubated with 20 μg/mL pHrodo Red *E. coli* BioParticles Conjugate with or without BzATP (300 μM) in ECS for 16 h at 37 °C. After washing, microglia were scraped and pelleted at 750 rpm for 5 min. The cell pellet was resuspended with 1× FAM-YVAD-FMK (Caspase-1 FLICA; Immunochemistry Technologies, Bloomington, MN, USA) and incubated at 37 °C for 90 min, with gentle mixing every 10 min. The microglia were pelleted, washed two times with Apoptosis Wash Buffer (Immunochemistry Technologies, Bloomington, MN), and resuspended in fresh normal extracellular solution. Cells were then added directly to an inverted epifluorescence microscope, pHrodo Red *E. coli* BioParticles were detected using 596/615 excitation and emission wavelengths, and Caspase-1 FLICA was detected using 488/510 excitation and emission wavelengths.

### Intracellular pH

Human microglia were plated onto collagen-coated coverslips for 24–72 h. The cells were incubated with or without BzATP (300 μM) for 16 h. To measure intracellular pH, the microglia were incubated with pHrodo Green AM Intracellular pH Indicator in ECS for 30 min at 37 °C. Cells were washed two times in ECS and mounted onto an inverted epifluorescence microscope. pHrodo Green was detected using 488/510 excitation and emission wavelengths.

### ELISA

Microglia cells were seeded in 96-well plates at a concentration of 7 × 10^4^ cells/well and incubated overnight. Cells were primed for 4, 6, or 24 h with 1 μg/ml or 10 μg/ml LPS in ECS and subsequently stimulated for 30 min with BzATP (300 μM) or ATP (5 mM) or 3 h with nigericin (20 μM) in 100 μl ECS at 37 °C. Microglia were also primed with 20 μg/mL pHrodo Red *E. coli* BioParticles Conjugate ± BzATP (300 μM) for 24 h. THP-1 monocytes were seeded in 96-well plates at a concentration of 5 × 10^4^ cells/well and incubated overnight. THP-1 monocytes were primed with 1 μg/mL LPS for 4 h followed by stimulation for 30 min with BzATP (300 μM) or 3 h with nigericin (20 μM) in 100 μl ECS at 37 °C. Supernatants and lysates (collected using lysis buffer containing the following (in mM): 150 NaCl, 25 HEPES, 5 EDTA, 1% Triton X-100, and 1× SIGMAFAST™ Protease Inhibitor Tablets (Sigma), pH 7.4) were collected and kept frozen at − 20 °C until analysis. Pro and mature IL-1β was evaluated with ELISA using the R&D Systems DuoSet kit (Cat # DY201-05) and the Invitrogen IL-1 beta Human Uncoated ELISA Kit (Cat # 88-7261-88) according to the manufacturer’s protocol. Total IL-18 was evaluated with ELISA using the R&D Systems Human Total IL-18/IL-1F4 Quantikine ELISA Kit (Cat # DL180) according to the manufacturer’s protocol. Developed plates were read on a Biotek Neo Alpha Plate Reader plate reader with Gen5 software (BioTek Instruments, Winooski, VT).

### Cell blebbing and annexin V binding

Human microglia were plated on an 8-well chambered slide (ibidi USA, Inc., Fitchburg, WI). The cells were incubated with or without BzATP (300 μM or 500 μM) or ATP (5 mM) for 15 min–24 h in ECS at 37 °C. After incubation, cells were analyzed for blebbing by imaging on a confocal microscope (Leica SP8 TCS STED 3X). Cells were also monitored for blebbing after exposure to BzATP or ATP with time-lapse confocal microscopy, with one frame taken every 15 s for 30 min. As a positive control, HEK-293T cells transfected with human P2X7R were plated onto chambered slides overnight. Slides were then placed onto the microscope stage, and a time course of blebbing was obtained by imaging cells after BzATP (500 μM) application for 30 min (1 frame every 15 s). Blebbing was quantified as the percentage of cells with blebs after BzATP stimulation. For the annexin V binding assay, microglia cells were grown on collagen-coated coverslips and treated with or without 20 μg/mL *E. coli* particles ± BzATP (300 μM) for 24 h in ECS. After treatment, the microglia were incubated with annexin V-FITC (BD Biosciences, San Jose, CA, USA) for 15 min at RT. Cells were subsequently analyzed using an inverted epifluorescence microscope where annexin V was detected with 488/510 excitation and emission wavelengths and analyzed using ImageJ software.

### Dye uptake

Microglia dye uptake was assayed using the dyes YO-PRO-1 (14 μM), YOYO-1 (10 μM) carboxyfluorescein (0.5 mM), Lucifer yellow (0.5 mM), or ethidium (10 μM). Cells were grown on collagen-coated coverslips and washed in normal ECS. Cells were incubated with 300 μM BzATP in ECS with and without the dyes for 15 min at 37 °C in a humidified atmosphere containing 5% CO_2_. In some experiments, cells were pre-incubated for 30 min with the following compounds: A804598 (20 μM), A438079 (50 μM), BX430 (10 μM), Tannic Acid (20 μM), DIDS (100 μM), carbenoxolone (20 μM), probenecid (5 mM), ^10^Panx1 peptide (300 μM). In Ca^2+^ free experiments, microglia were pre-incubated with BAPTA-AM (10 μM) for 60 min followed by addition of YO-PRO-1 ± BzATP in Ca^2+^ free ECS containing EDTA (1 mM). In experiments using nigericin, cells were incubated with YO-PRO-1 and nigericin (20 μM) for 30 min at 37 °C. In Cl^−^-free experiments, microglia cells were treated in solution containing the following (in mM): 140 Na gluconate, 5 K gluconate, 5.5 glucose, and 10 HEPES, pH 7.4. In the time-course experiments, YO-PRO-1 and ethidium fluorescence were measured after BzATP stimulation every 30 s for 30 min. The dye uptake was measured by fluorescence microscopy using an inverted epifluorescence microscope (Eclipse TE2000, Nikon) fitted with a CCD camera. YO-PRO-1, YOYO-1, carboxyfluorescein, and Lucifer yellow were measured using 488/510 excitation and emission wavelengths. Ethidium was measured using 596/615 excitation and emission wavelengths.

### LDH release

Microglia were seeded in 96-well plates at a concentration of 7 × 10^4^ cells/well and incubated overnight. Microglia were then treated with BzATP (300 μM), ATP (5 mM), or nigericin (20 μM) for 30 min at 37 °C. Untreated microglia served as the negative control while lysed cells served as the positive control. Fifty microliters of cell supernatant was collected and used to detect LDH activity with the CytoTox96 Non-Radioactive Cytotoxicity Kit (Promega, Madison, WI, USA) according to the manufacturer’s instructions.

### Imaging analysis

In all fluorescence microscopy observations, cells were also observed with clear field illumination and 25–150 cells were present in each microscope field studied. Imaging acquisition and data analysis were performed with the software package ImageJ. We used the following formula to measure the corrected total cell fluorescence (CTCF) in relative fluorescence units (RFU):

$$ \mathrm{CTCF}=\mathrm{whole}\ \mathrm{cell}\ \mathrm{signal}-\left(\mathrm{area}\times \mathrm{background}\ \mathrm{signal}\right) $$

where “whole cell signal” equals the sum of the intensity of the pixels for one cell, area equals the number of pixels defining the cell, and “background signal” equals the average signal per pixel for a region devoid of cells but close to the cell of interest [30].

### Statistical analysis

Data were analyzed using GraphPad Prism and reported as mean ± s.e.m. Student’s *t* test (for paired or unpaired samples as appropriate) and analysis of variance with Tukey post-test were used for statistical analysis. *p* < 0.05 was accepted as a significant difference.

## Results

In contrast to sexually dimorphic patterns of purinergic receptor mRNA expression in mouse microglia [31], we saw no gender-based differences in the responses of cultured human microglia to ATP. Therefore, we obtained a similar number of results from male and female donor cells for each protocol reported here and pooled the results for statistical analyses of the effect of drugs and treatments.

### Gene expression suggests that cultured human microglia adopt a phagocytotic phenotype

**Fig. 1:**
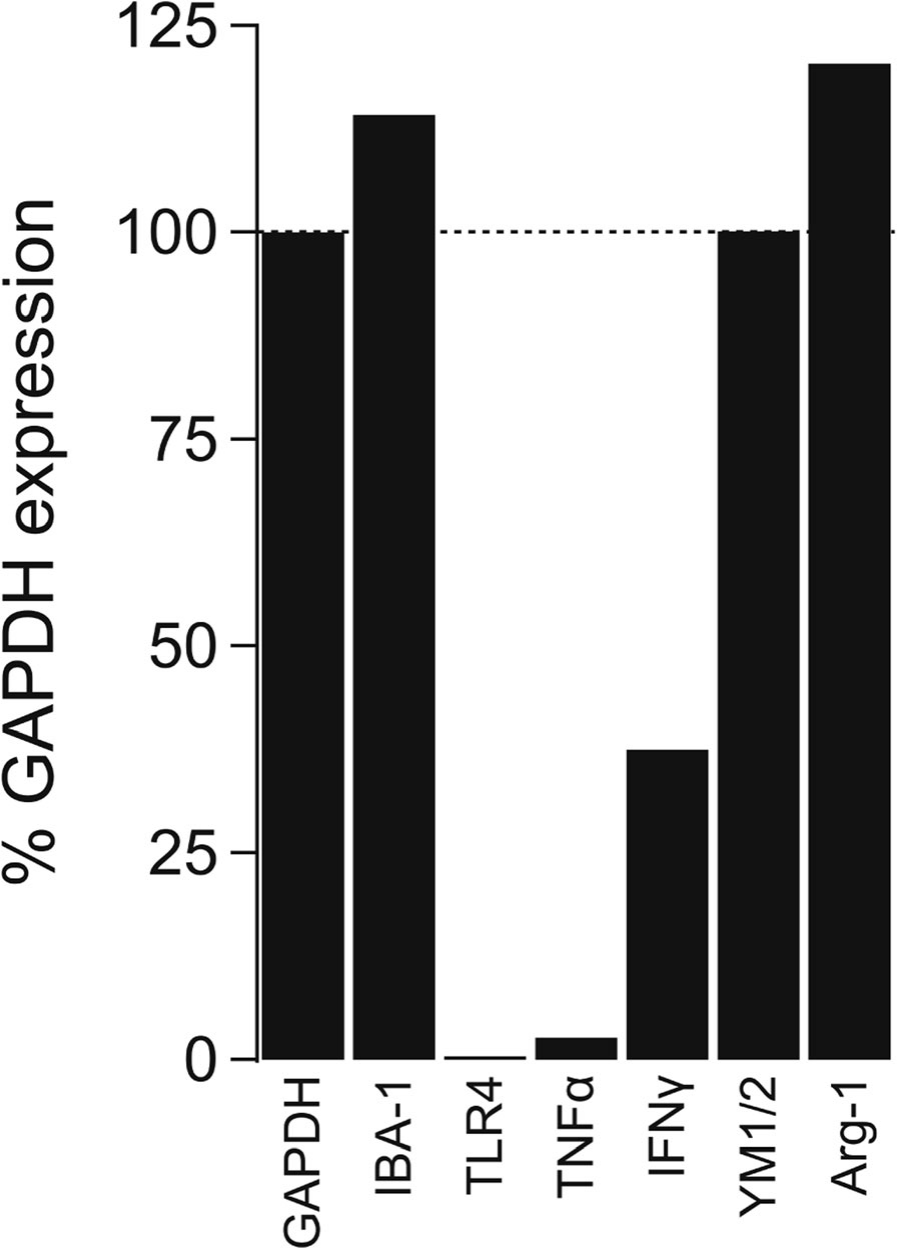
Gene expression in cultured human microglia. The bar graph shows gene expression normalized to GAPDH

Real-time quantitative PCR was performed to check for the presence of genes associated with different activation states (Fig. 1). First, we sought to confirm the identity of the cultured cells by measuring expression of IBA-1. IBA-1 is a cytoplasmic protein that is exclusively expressed in brain microglia. We saw abundant expression in our cultured cells, confirming their identity. Then, we focused on three genes (interferon gamma (IFNγ), toll-like receptor 4 (TLR4), and tumor necrosis factor alpha (TNFα)) to determine the phenotype of the cultured microglia. Microglia are highly reactive cells that have the ability to express both classical pro-inflammatory and phagocytotic markers. TLR4 is a pattern recognition receptor for LPS, which triggers transcription of both IL-1β and IL-18 precursors. The fact that we saw no measurable TLR4 gene expression in our cultured human microglia suggests an anti-inflammatory phenotype. Next, we measured expression of two additional genes, IFNγ and TNFα, that are associated with the pro-inflammatory phenotype and again saw low expression. Finally, we measured expression of genes typically associated with the phagocytosis phenotype and saw robust expression of Arg-1 and YM1/2. Altogether, these data suggest that primary human microglia adopt an anti-inflammatory and phagocytotic state under the culture conditions used in our experiments.

### Human microglia express functional P2X7 receptors

**Fig. 2:**
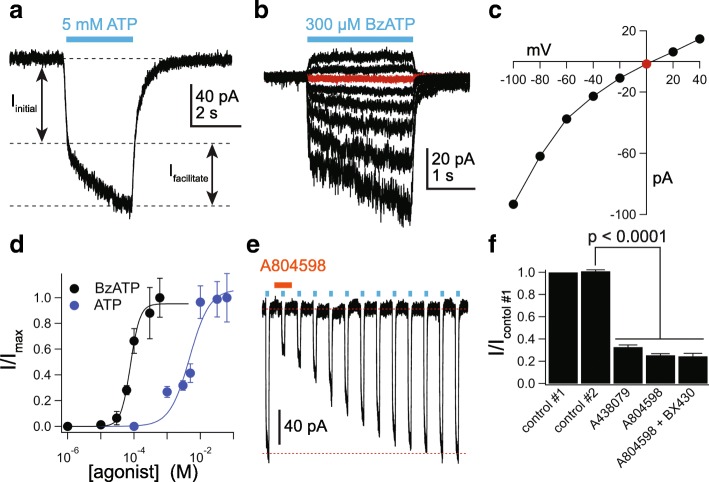
Human microglia express functional P2X7Rs. **a** Representative tracing of a whole-cell current (holding voltage = − 60 mV) activated by 5 mM ATP in a free-floating primary adult human microglia. The current displayed two distinct phases: the first (*I*_initial_) is the immediate effect of agonist application and the second (*I*_facilitate_) reflects the time-dependent facilitation. **b** Representative tracing of a microglial current activated by 300 μM BzATP at different holding potentials (− 100 to + 40 mV). **c** The current-voltage (*I*-*V*) relation obtained from the peak current amplitudes of the microglia cell shown in panel **b**. The reversal potential is indicated by the red symbol. **d** Concentration-response curves for ATP and BzATP in human microglia. Symbols represent means, and vertical lines are s.e.m. of the normalized current density from 5 to 25 cells. Sigmoidal curve is the best fit obtained with a four-parameter logistic function. **e** Representative inhibition and recovery after a 2-min incubation in 20 μM of the P2X7R antagonist A804598. The concentration of ATP was 5 mM. **f** ATP-gated (5 mM) current amplitude as fraction of control #1: the current amplitude before antagonist application. Control #2 represents the *I*_ATP_ measured after a 2-min incubation in control ECS solution before antagonists were applied. The fact that the amplitude of control #2 is not different from that of control #1 shows that the current is fully facilitated before application of antagonist. Current is significantly inhibited by the P2X7R antagonists A438079 (50 μM, *n* = 15; *p* < 0.0001; paired *t* test) and A804598 (20 μM, *n* = 17; *p* < 0.0001; paired *t* test). The current that remains after inhibition by A804598 is unaffected by co-application of the P2X4R antagonist BX430 (5 μM, *n* = 10; no significant difference between A804598 alone). All antagonists were applied 2 min before ATP application

We used whole-cell voltage-clamp electrophysiology to explore the presence of functional P2X receptors in cultured human microglia. ATP (5 mM) induced a biphasic inward current at a holding potential of − 60 mV whose amplitude progressively grew during a 3-s application (Fig. 2a) This “facilitating” phenotype closely matched that predicted for a uniform population of P2X7Rs, which are the purinergic channels most closely associated with DAMP-mediated responses [32]. The agonist-gated peak current amplitude varied as a function of the holding potential and reversed direction at 0 mV (Fig. 2b, c), as expected for non-selective cation current through an ionotropic P2X receptor [33]. The average reversal potential measured from 13 cells was 0.9 ± 1.5 mV. We saw no evidence of an initial phase of a rapidly desensitizing inward current expected from P2X1Rs and P2X3Rs, and we failed to record membrane current in response to 100 μM ATP thus ruling out P2X2Rs, P2X4Rs, and P2X5Rs.

We measured the dependence of the current amplitude on the concentration of ATP and the higher potency agonist, BzATP, and calculated EC_50_s of 4.7 mM and 60.8 μM (Fig. 2d), respectively, which are close to those previously reported for recombinant human P2X7Rs determined in the presence of a physiological concentration of Ca^2+^ [34, 35]. We then studied the effects of selective receptor antagonists using 5 mM ATP as agonist. We used this concentration of ATP because it evoked currents with easily measured peak amplitudes and produced full facilitation in a reasonable amount of time. We found that inward current gated by 5 mM ATP was inhibited by preincubating the cells for 2 min in the competitive P2X7R antagonist A438079 (50 μM) [36] and the non-competitive P2X7R antagonist A804598 (20 μM) [37] (Fig. 2e, f). The inhibitions were highly significant but incomplete at the concentrations of ATP and antagonists tested; we did not test higher antagonist concentrations prone to non-selective effects. The remaining ATP-gated current was not inhibited by the selective P2X4R antagonist BX430 [38], confirming the absence of a contribution of P2X4Rs (Fig. 2f). Further, we pre-incubated cells in 10 μM ivermectin, a positive allosteric modulator of P2X4Rs [39], and continued to see no current in response to 100 μM ATP (Additional file 1: Figure S1a), providing additional evidence that cultured human microglia do not express functional P2X4Rs in their cell surface membrane. In contrast, we recorded significant potentiation by ivermectin of the current gated by a saturating concentration of ATP (10 mM; Additional file 1: Figure S1a, b), which supports the hypothesis that ivermectin is also a positive allosteric modulator of human P2X7Rs as previously reported for monocyte-derived human macrophages [40].

**Fig. 3:**
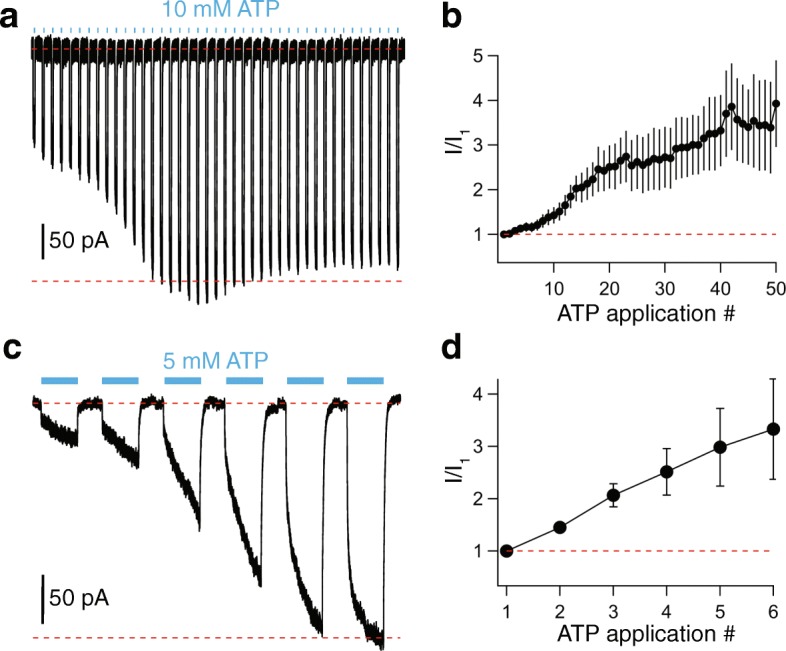
ATP-gated currents facilitate. **a** Representative tracing of facilitating inward currents recorded from successive applications of 10 mM ATP (3 s duration) separated by 15-s intervals, from a holding potential of − 60 mV. **b** Time course of facilitation measured as the fraction of current recorded at the end of each successive 3-s ATP application over the initial current (*n* = 25 cells). **c** Tracing of microglial facilitation recorded from successive applications of 5 mM ATP (15 s duration) separated by 30-s intervals, from a holding potential of − 60 mV. **d** Time course of facilitation measured as the fraction of current recorded at the end of each successive 15-s ATP application over the initial current (*n* = 7 cells)

Next, we took a closer look at the current facilitation which is a hallmark property of P2X7Rs [27]. We applied ATP once every 15 s (Fig. 3a) and saw a progressive increase in peak inward current amplitude that grew fourfold over the course of 60 3-s applications (Fig. 3b). As expected for a P2X7R-mediated response [28], increasing the length of agonist exposure in a single volley from 3 to 15 s produced a similar degree of facilitation with a fewer number of repetitive ATP applications (Fig. 3c, d).

Taken together, the shape of the current, the reversal potential near 0 mV, the overall insensitivity to ATP and BzATP as indicated by their moderately high EC_50_ values, the markedly higher potency of BzATP by comparison to ATP, the inhibition by P2X7R selective antagonists, the lack of effect of ivermectin on micromolar concentrations of ATP, and the pronounced current facilitation all suggest that cultured human microglia express a uniform population of functional P2X7Rs.

### Activation of P2X7Rs increases [Ca^2+^]_i_

**Fig. 4:**
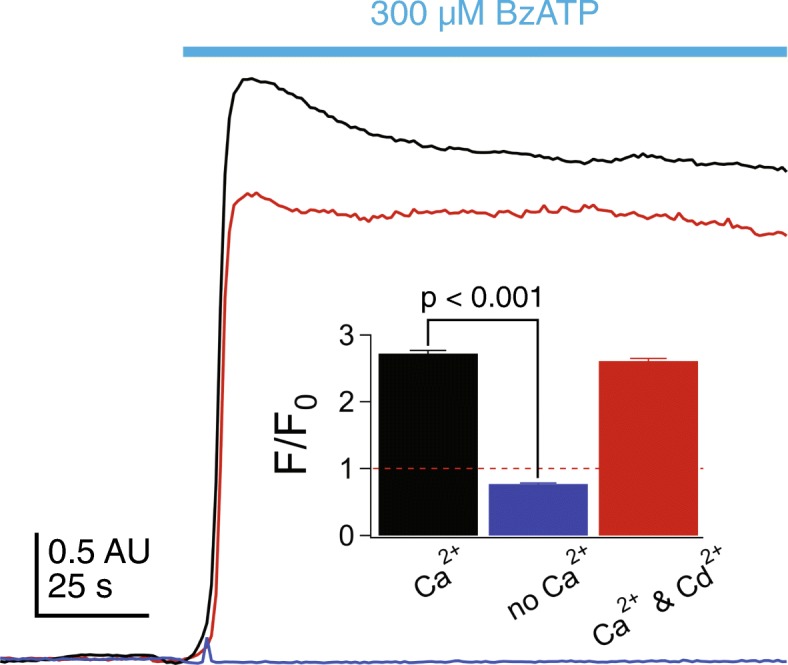
BzATP increases [Ca^2+^]_i_ by gating P2X7Rs. Microglia grown on coverslips were incubated with 5 μM Fluo-4-AM for 30 min, followed by 30 min in dye-free medium to allow de-esterification. 300 μM BzATP application (black trace) stimulated an immediate rise in fluorescence that plateaued during constant agonist exposure. No change in [Ca^2+^]_i_ was measured in the absence of extracellular Ca^2+^ (blue trace). BzATP-induced increase in [Ca^2+^]_i_ fluorescence was unaffected by addition of the non-selective Ca^2+^ channel blocker, cadmium (200 μM) (red trace). Inset shows quantification of fluorescence changes over time, measured as the fold change in fluorescence over baseline after background subtraction (*F*/*F*_0_) for 7–11 separate experiments for each paradigm. Significance was determined using ANOVA

Ca^2+^ triggers a number of downstream signaling pathways in glia [41]. To determine if the P2X7R is a significant source of Ca^2+^ in cultured adult human microglia, we visualized changes in [Ca^2+^]_i_ using Fluo-4-AM. Microglia grown on coverslips were incubated with 5 μM Fluo-4-AM for 30 min, followed by 30 min in dye-free medium to allow de-esterification. Unstimulated microglia bathed in normal extracellular medium showed a dim but measurable level of fluorescence (excitation 494 nm: emission 514 nm), indicating a low resting [Ca^2+^]_i_. Fluorescence rose quickly to a plateau in response to 300 μM BzATP (black trace, Fig. 4) signifying a persistent rise in [Ca^2+^]_i_ during constant agonist exposure. The increase in [Ca^2+^]_i_ was unaffected by addition of the non-selective Ca^2+^ channel blocker, cadmium (200 μM), to the extracellular medium (red trace). In contrast, removing extracellular Ca^2+^ completely abolished the fluorescence change (blue trace). Taken together, these results show that Ca^2+^ current through the P2X7R pore is an absolute requirement for the ATP-gated change in [Ca^2+^]_i_ in cultured adult human microglia.

### LPS stimulation of human microglia enhances P2X7R current density

**Fig. 5:**
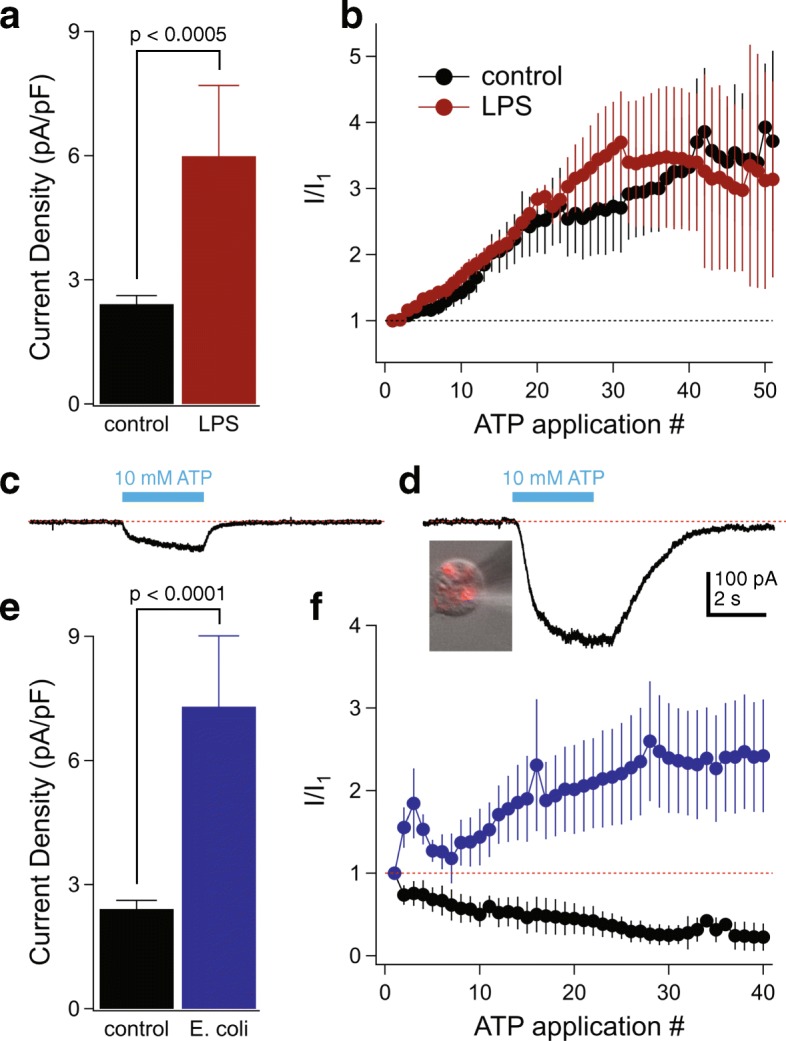
Microglial P2X7Rs are dynamically regulated by inflammatory stimuli. **a** The mean current density (pA/pF) of the initial ATP application (10 mM, 3 s) measured in microglia treated with LPS (1 μg/mL, 12–24 h) was 2.5-fold greater than untreated control cells (*n* = 54 control cells, *n* = 14 LPS cells; *p* < 0.0005; unpaired *t* test). **b** Comparison of facilitation time course between control and LPS-treated cells measured as the fraction of current recorded at the end of each successive 3-s ATP application over the initial current (*n* = 25 control cells, *n* = 7 LPS cells). **c** Representative patch-clamp recording of a 3-s ATP-gated current (10 mM) recorded from an untreated (non-phagocytic) human microglia cell. **d** Example of a microglia cell that had phagocytosed red-fluorescent *E. coli* bioparticles (inset photo); patch-clamp recording from this cell showed significantly increased 10 mM ATP-mediated current compared to untreated microglia. Panels **c** and **d** share a common scale bar. **e** The mean current density (pA/pF) of the initial ATP application (10 mM, 3 s) measured from phagocytic microglia was threefold greater than untreated control cells (*n* = 54 control cells, *n* = 23 phagocytic cells; significant enhancement by *E. coli* ingestion *p* < 0.0001; unpaired *t* test). **f** Time course of facilitation (blue symbols) or rundown (black symbols) of phagocytic microglia currents measured as the fraction of current recorded at the end of each successive 3-s ATP (10 mM) application over the initial current (*n* = 10 facilitation cells, *n* = 11 run-down cells)

LPS is a pro-inflammatory stimulus that causes a 2 to 4-fold increase in the P2X4R-mediated response of immortalized mouse microglial cells without affecting P2X7R currents [42, 43]. We sought to determine if a similar pattern occurs in humans. Overnight treatment with LPS caused a 2.5-fold increase in peak current amplitude (control, 2.4 ± 0.2 pA/pF, *n* = 54; LPS, 6.0 ± 1.7 pA/pF, *n* = 14) gated by 10 mM of ATP (Fig. 5a) but had no effect on the time course of facilitation (Fig. 5b). However, LPS did not result in a change in the shape of the ATP-gated current, and we saw no indication of a fast spike of current as expected from activation of a population of P2X4Rs. Further, LPS did not render cultured human microglia sensitive to a concentration of ATP (100 μM) sufficient to maximally activate P2X4Rs, suggesting that LPS does not upregulate this receptor in humans. The ability of LPS to increase P2X7R-mediated current density is interesting in light of the lack of measurable expression of the classical LPS receptor, TLR4 (see Fig. 1), suggesting the presence of non-canonical LPS receptors for this action in cultured human microglia [44].

### Human microglial P2X7Rs are dynamically regulated by *E. coli* phagocytosis

Because phagocytosis is a vital function of microglia during postnatal development [1], we sought to determine if human microglia retain this function in culture. Towards this end, we incubated cells for 16–24 h in ECS containing pHrodo Red *E. coli* bioparticles that fluoresce in the acidic environment of a phagosome. We saw strong red fluorescence inside cells, thus demonstrating phagocytosis (inset, Fig. 5d). We measured ATP-currents in fluorescing cells and found that phagocytosis was accompanied by a 3-fold enhancement of peak current density measured from the first application of 10 mM ATP by comparison to cells not exposed to the *E. coli* bioparticles (Fig. 5c–e); these enhanced currents remained cation non-selective (Additional file 2: Figure S2a). Surprisingly, the phagocytic microglia displayed two distinct current profiles with repetitive activation. Some cells showed a typical pattern of current facilitation while others (11 of 21 for the data of Fig. 5d) showed a gradual decrease in peak current density with repetitive applications (rundown) that eventually plateaued to a steady-state (Fig. 5f). A typical recording of this current rundown is shown in Additional file 2: Figure S2b. At present, the reason why some cells show facilitation while others show rundown is unknown, as we saw no other obvious differences in the behavior of these cells. HEK293 cells transiently expressing rat P2X7Rs occasionally rundown with repetitive applications of ATP as a specific tyrosine in the pore-lining transmembrane domain is dephosphorylated [45], and it is possible that phagocytosis of *E. coli* in human microglia in some cases stimulates recruitment of a phosphatase to the P2X7R signaling complex.

In keeping with our previous results using LPS, cultured human microglia exposed to *E. coli* bioparticles remained insensitive to 100 μM ATP, indicating that phagocytosis did not prime cells for ATP-gated P2X4R current.

### P2X7Rs decrease phagocytosis of *E. coli* particles

**Fig. 6:**
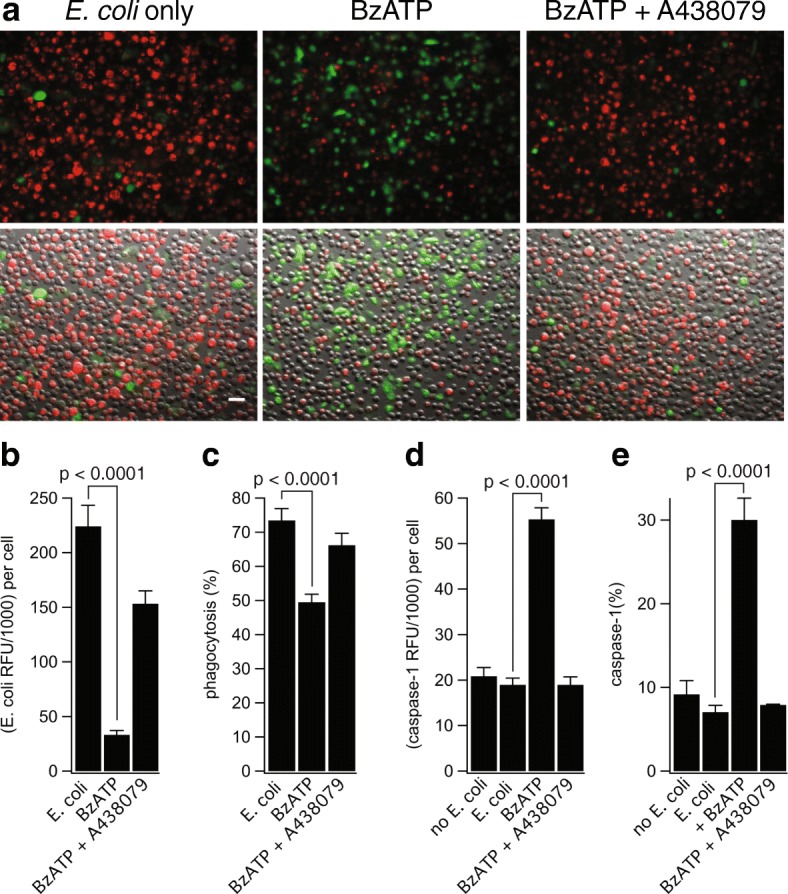
P2X7R decreases phagocytosis independently of IL-1β release. **a** Representative fluorescence and clear field images of human microglia incubated with *E. coli* bioparticles (red) and treated with or without BzATP (300 μM) ± A438079 (50 μM) followed by staining for activated caspase-1 (green). Scale bar: 20 μm. **b** Human microglia displayed strong spontaneous ingestion of *E. coli* (224.3 ± 19.1 RFU), and BzATP treatment resulted in strong inhibition of *E. coli* ingestion (33.37 ± 3.82 RFU). Co-incubation of BzATP with A438079 significantly increased the amount of *E. coli* ingestion (153.4 ± 11.8 RFU). The histograms show mean ± s.e.m. for “*n*” of 38–40 cells (*p* < 0.0001; ANOVA). **c** Human microglia displayed strong spontaneous phagocytic capacity (73.5% of cells were *E. coli* positive) and stimulation of the P2X7R with BzATP resulted in strong inhibition of this percentage (49.6%). Normal levels of phagocytosis were restored by antagonizing the P2X7R with A438079 (66.2%). The histograms show mean ± s.e.m. for an “*n*” of 7–8 separate experiments (*p* < 0.0001; ANOVA). **d**, **e** Activated caspase-1 RFU (**d**) and percentage of microglia with activated caspase-1 (**e**) were measured after *E. coli* treatment. Microglia displayed low levels of caspase-1 activation when treated with *E. coli* alone (18.37 ± 1.46 RFU, 7.1% of cells), and stimulation with BzATP resulted in strong caspase-1 activation (55.35 ± 2.50 RFU, 30.1% of cells). Normal levels of caspase-1 activation were restored by co-incubation of BzATP with A438079 (18.98 ± 1.73 RFU, 7.9% of cells). The histograms show mean ± s.e.m. for an “*n*” of 5 separate experiments (*p* < 0.0001; ANOVA)

P2X7Rs act as scavenger receptors that promote phagocytosis by directly binding apoptotic cells and bacteria to their extracellular domain in the absence of receptor stimulation by ATP [46]. The scavenger receptor function of the P2X7R depends on its tight association with non-muscle myosin IIA, which only occurs in the resting conformation [47]. Upon activation of the P2X7R with ATP, non-muscle myosin is dissociated from this complex and phagocytosis is significantly reduced [48]. To determine if activation of P2X7Rs blocks phagocytosis in cultured human microglia, we measured phagocytosis of red-fluorescent *E. coli* bioparticles in the absence and presence of BzATP (300 μM). We measured significant inhibition of phagocytosis that was prevented by pre-incubation with the P2X7R antagonist A438079 (50 μM; Fig. 6a–c). The ability of BzATP to inhibit pH-sensitive *E. coli* fluorescence was not caused by a global change in cytoplasmic pH which remained unchanged after application of BzATP (Additional file 3: Figure S3a). Thus, our data support the contention that unstimulated P2X7Rs act as scavenger receptors in human tissues.

### P2X7Rs are unable to trigger IL-1β or IL-18 release

In human microglia, we measured strong activation of caspase-1, the enzyme responsible for maturation of IL-1β and IL-18, by 300 μM BzATP which was blocked by antagonizing the P2X7R (Fig. 6a, d, e). Surprisingly, the *E. coli*-positive phagocytic microglia were entirely negative for activated caspase-1 (i.e., co-localization was never observed between the two signals; see Fig. 6a). These results suggest that activation of the P2X7R causes human microglia to limit anti-inflammatory pathways that support engulfment of pathogens and instead promote inflammatory pathways that activate caspase-1. However, despite activation of caspase-1, we were unable to detect either production or release of IL-1β from cultured human microglia primed with LPS or *E. coli* particles and stimulated with BzATP, ATP, or the K^+^/H^+^ ionophore nigericin. As a positive control, we detected robust release of IL-1β from the immortalized human monocyte cell line (THP-1 cells) primed with LPS and stimulated with either BzATP or nigericin (Additional file 3: Figure S3b). Since caspase-1 is also responsible for maturation of IL-18, we tested whether activated microglia were capable of releasing this pro-inflammatory cytokine. Interestingly, microglia primed with LPS and activated with either BzATP or nigericin were unable to stimulate the release of IL-18, despite having intracellular IL-18 present in their lysates (Additional file 3: Figure S3c). Previous reports in human monocytes found that the homozygous ^496^Glu to Ala (^496^Ala/Ala) single nucleotide polymorphism (SNP) in the P2X7R impairs ATP-induced IL-1β and IL-18 release [49, 50]. We used an external vendor (Novogene Corp, Chula Vista, CA) to perform whole exosome sequencing to determine if either of our donors carry this mutation; both were heterozygous at ^496^Glu/Ala. Published work shows that human monocytes from heterozygotic donors phagocytose fluorescent beads and undergo ATP-evoked membrane permeabilization, suggesting that ^496^Glu/Ala monocytes retain the wild-type phenotype [51]. We found that ATP triggers inward current blocked by P2X7R antagonists in microglia from heterozygotes (see Fig. 2f), demonstrating functional P2X7Rs in the plasma membrane of our cultured cells. Thus, we conclude that the inability of ATP to trigger release of inflammatory cytokines from cultured human microglia does not result from a loss-of-function P2X7R phenotype. Further, our data show that P2X7R-mediated caspase-1 activation in cultured human microglia does not trigger the release of IL-1β or IL-18.

### The P2X7R does not activate human microglial blebbing

In cultured murine microglia, activation of P2X7Rs causes blebbing of the membrane, leading to shedding of microvesicles that contain phosphatidylserine on their outer leaflet and IL-1β inside [52]. We used confocal microscopy to image changes in the cell surface membrane and found that activation of the P2X7R with BzATP does not cause bleb formation in human microglia (Additional file 4: Video S1). As a positive control, we identified robust BzATP-induced blebbing in HEK293T cells transfected with human P2X7Rs (Additional file 3: Figure S3d). Moreover, microglia cells did not undergo phosphatidylserine switch in the presence of BzATP, even after stimulation with *E. coli* particles (Additional file 3: Figure S3e). Thus, the inability of BzATP to stimulate blebbing and phosphatidylserine flip suggest that P2X7Rs do not trigger apoptosis of cultured human microglia cells.

### P2X7 receptors permeabilize cultured human microglia

**Fig. 7:**
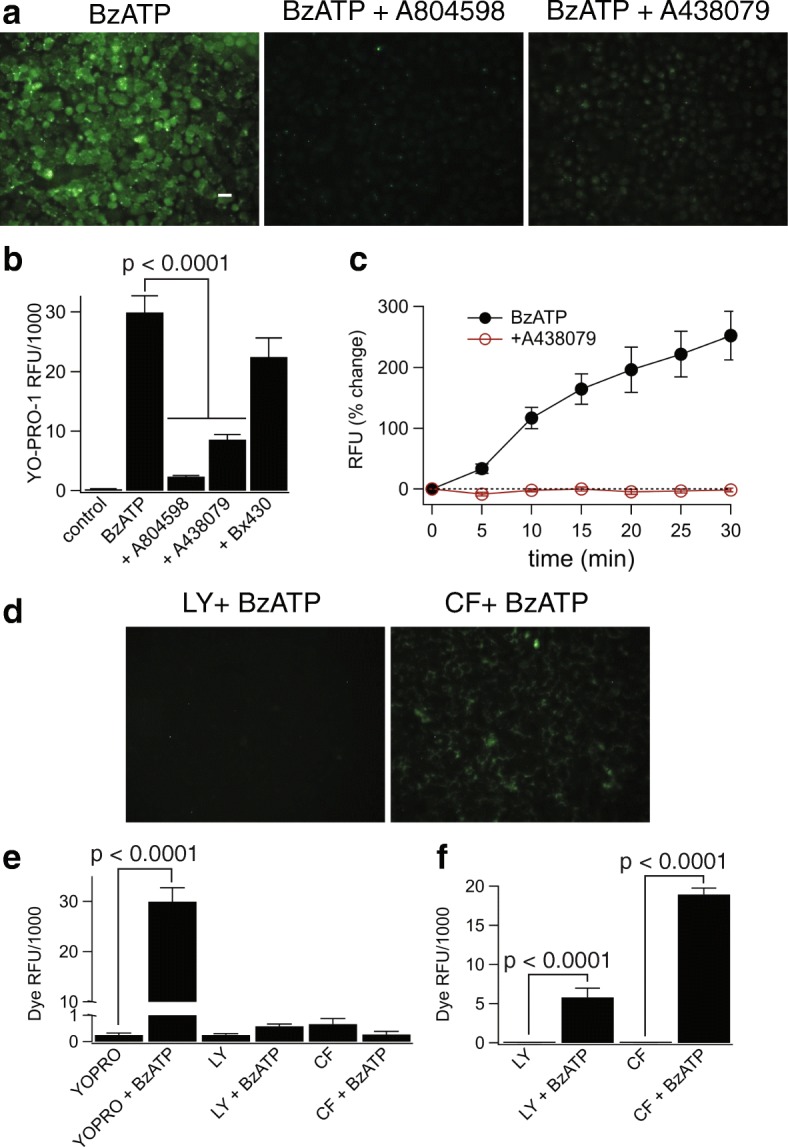
Stimulation of the P2X7R causes robust permeabilization of human microglia to exclusively cationic dyes. **a** Stimulation of the P2X7R with BzATP (300 μM) induced the uptake of the cationic dye YO-PRO-1 in human microglia. Representative fluorescence images of cells incubated for 15 min in the presence of 300 μM ATP +/− the antagonists A804598 (20 μM) or A438079 (50 μM). Each antagonist was pre-incubated for 30 min. Scale bar: 20 μm. **b** Quantitative comparison of YO-PRO-1 uptake by microglia. Cells were incubated for 15 min with YO-PRO-1 and 300 μM BzATP in the presence of various inhibitors: BX430 (10 μM), A438079 (50 μM), and A804598 (20 μM), *n* = 4 separate experiments; there was significant inhibition by P2X7R antagonists (*p* < 0.0001; ANOVA). **c** Time course of YO-PRO-1 uptake shows the average s.e.m. of fluorescence intensity over time measured from individual cells within a single field of view after application of 300 μM BzATP +/− A438079 (50 μM). **d**, **e** Activation of the P2X7R with BzATP (300 μM) does not induce the uptake of the anionic dyes Lucifer yellow (Lucifer yellow, 0.5 mM) or carboxyfluorescein (CF, 0.5 mM) in human microglia. **d** Example fluorescence images of cells incubated in the presence of 300 μM ATP with the anionic dyes Lucifer yellow or CF. **e** Quantitative comparison of anionic dye uptake by microglia. Cells were incubated for 15 min with Lucifer yellow or CF in the presence of BzATP (300 μM). There is no significant difference between dye uptake in control and treated cells (*n* = 4 separate experiments). **f** Unlike human microglia, J774A.1 mouse macrophages display strong uptake of anionic dyes. Mouse macrophages were incubated for 15 min with Lucifer yellow (0.5 mM) or CF (0.5 mM) in the presence of ATP (5 mM), *n* = 3 separate experiments; there was significant difference of dye RFU after ATP treatment (*p* < 0.0001; unpaired *t* test)

The immediate effect of activating P2X7Rs is the increased flow of Na^+^, K^+^, and Ca^2+^ across the cell surface membrane. The net result is depolarizing current at physiological membrane potentials [33]. A second effect occurs as the membrane becomes permeable to larger molecules (< 900 Da), initiating a number of downstream sequela including inflammation and cell death [53, 54]. To determine if cultured human microglia undergo permeabilization, we measured the change in whole-cell fluorescence that accompanies uptake of the large (630 Da) carbocyanine nucleic acid stain YO-PRO-1. We measured YO-PRO-1 fluorescence before and during 15–30-min applications of 300 μM BzATP and saw a time-dependent increase in total cell fluorescence (Fig. 6a–c). We noted similar results using ethidium, another nucleic acid stain (394 Da; Additional file 5: Figure S4a, b). YO-PRO-1 uptake was strongly inhibited by the P2X7R selective blockers A438079 (50 μM) and A804598 (20 μM) but not by the P2X4R antagonist BX430 (10 μM) (Fig. 7a–c). Activated P2X7Rs were unable to permeate the larger cationic dye YOYO-1 (1270 Da, Additional file 5: Figure S4c), indicating that the dye uptake pathway had a molecular size cutoff point. Moreover, the dye uptake pathway was non-lytic as neither BzATP nor ATP stimulated LDH release from the microglia (Additional file 5: Figure S4d).

### The permeabilization pathway is cation selective

ATP triggers uptake of cationic and anionic dyes through independent pathways in Raw 264.7 cells, a macrophage-derived mouse cell line expressing the P2X7R [55, 56]. YO-PRO-1 and ethidium are cations, and as noted above, we measured robust uptake of both dyes in cultured human microglia. In contrast, 300 μM BzATP did not trigger significant uptake of the fluorescent anions Lucifer yellow (457 Da; 500 μM) or carboxyfluorescein (376 Da; 500 μM) (Fig. 7d, e). As a positive control [57], we measured robust ATP-induced Lucifer yellow and carboxyfluorescein uptake in J774 mouse macrophages (Fig. 7f). Taken together, our data show that cultured human microglia express an ATP-dependent pathway for the passage of large organic cations across the cell surface membrane but lack the conduit for large anions present in murine immune cells.

### Dye uptake is independent of pannexin-1 channels

**Fig. 8:**
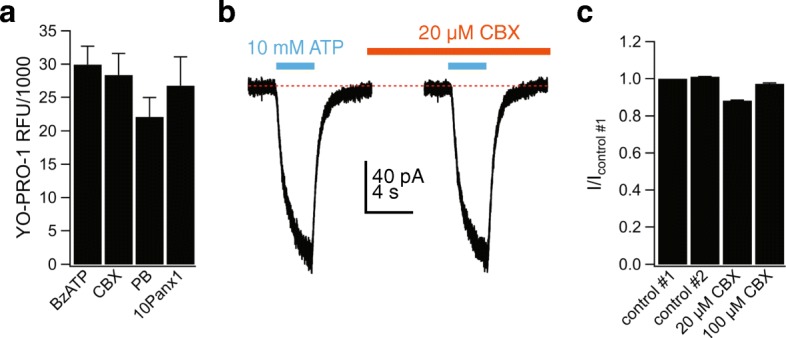
Pannexin-1 is not involved in permeabilization of human microglia. **a** The uptake of YO-PRO-1 into human microglia is independent of pannexin-1 channels. Microglia were pre-incubated for 30 min with the pannexin-1 inhibitors carbenoxolone (CBX, 20 μM), probenecid (PB, 5 mM), or ^10^panx (300 μM) and subsequently assessed for YO-PRO-1 uptake after 15 min in the presence of BzATP (300 μM). None of the antagonists had a significant effect on dye uptake (*n* = 3 separate experiments). **b** Representative patch-clamp current tracing of microglia stimulated with ATP (10 mM, 3 s) before and after 2-min incubation with CBX (20 μM). **c** Current amplitude (as fraction of control #1: the current amplitude before antagonist application) induced by ATP (5 mM) after 2-min incubation with CBX (20 or 100 μM) was not significantly different from control #2. Control #2 represents the *I*_ATP_ measured after 2-min incubation in control ECS solution

The identity of the cation-selective pathway responsible for permeabilization is controversial. Strong evidence support direct passage of YO-PRO-1 through the P2X7R pore [53, 58], while other reports suggest the involvement of downstream proteins [59–61]. The most prominent candidate is pannexin-1, a hemi-channel permeable to YO-PRO-1 [62] and implicated in permeabilization of murine microglial BV-2 cells [63] and release of IL-1β from murine macrophages [64]. We measured whole-cell fluorescence in the presence of a range of pannexin-1 inhibitors (20 μM carbenoxolone, 5 mM probenecid, and 300 μM ^10^Panx inhibitory peptide) and saw no effect of these antagonists on the robust uptake of YO-PRO-1 by human microglia incubated in 300 μM BzATP for 15 min (Fig. 8a). Moreover, carbenoxolone had no effect on the peak amplitude of the fully facilitated ATP-gated current measured using electrophysiology (Fig. 8b, c). Collectively, our results show that both ATP-induced membrane permeabilization and cation channel activity are independent of pannexin-1 channels in cultured human microglia.

### Membrane permeabilization is attenuated by Cl^−^ channel antagonists

**Fig. 9:**
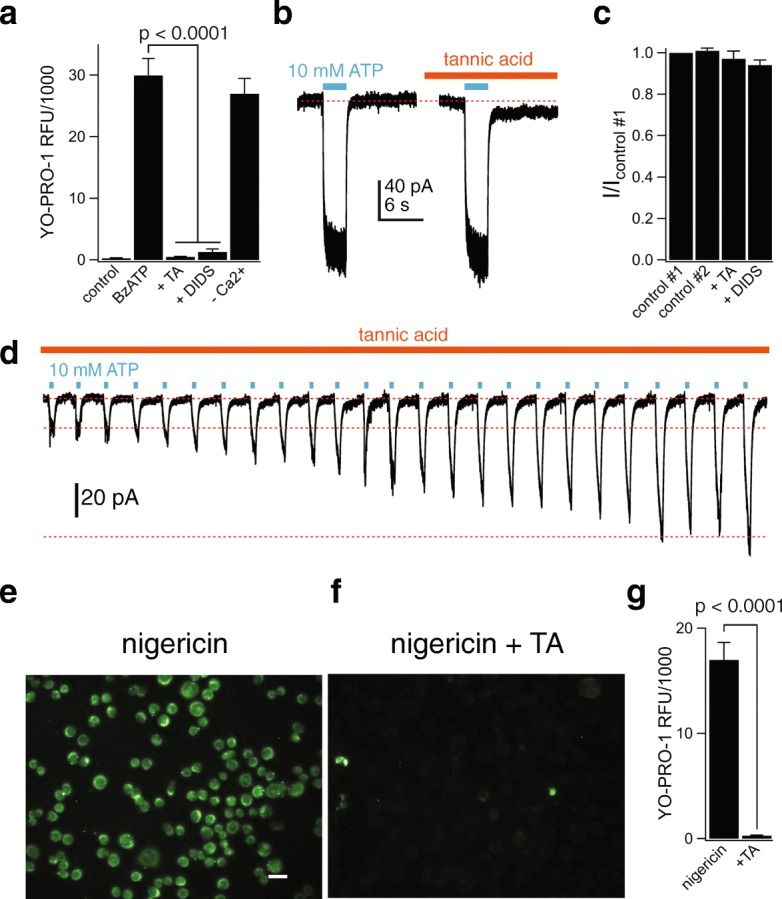
P2X7R-mediated activation of a chloride channel is responsible for human microglial permeabilization. **a** Microglia were pre-incubated for 30 min with the non-selective chloride channel inhibitors tannic acid (TA, 20 μM) or DIDS (100 μM), and YO-PRO-1 uptake was measured after 15 min in the presence of BzATP (300 μM) in normal extracellular solution. Both antagonists significantly inhibited dye uptake; *p* < 0.0001; ANOVA. The independence of YO-PRO-1 uptake on calcium flux was assessed by loading the cells with 10 μM BAPTA-AM and measuring dye uptake in Ca^2+^-free extracellular solution with 1 mM EDTA (-Ca^2+^; no significant difference from control). YO-PRO uptake in Cl^−^-free solution (-Cl^−^; where extracellular Cl^−^ was replaced with gluconate) was not significantly different from control. *n* = 4–8 separate experiments for all conditions. **b** Representative tracing of whole-cell currents (holding voltage = − 60 mV) activated by 10 mM ATP in primary adult human microglia before and after 2-min incubation with TA. **c** Current amplitude (as fraction of control #1: the current amplitude before antagonist application) induced by ATP (10 mM) in the presence of tannic acid (20 μM, *n* = 12 cells) or DIDS (100 μM, *n* = 7 cells) was not significantly different from control #2. Control #2 represents the *I*_ATP_ measured after 2-min incubation in control ECS solution. **d** Representative tracing of microglial 10 mM ATP-gated current that continues to facilitate even in the constant presence of TA (20 μM). **e**, **f** Fluorescence images of YO-PRO uptake in microglia incubated for 30 min in the presence of 20 μM nigericin (**e**) + 20 μM TA (**f**). Scale bar: 20 μm. **g** Quantification of nigericin-stimulated YO-PRO-1 uptake by microglia reveals significant inhibition of dye uptake by the chloride channel inhibitor TA, *n* = 3 separate experiments, significant inhibition by TA (*p* < 0.0001; unpaired *t* test)

A recent report suggests that anoctamin 6 (ANO6; also known as TMEM16F), a Ca^2+^-dependent phospholipid scramblase and Ca^2+^-activated Cl^−^ channel, is required for ATP-induced dye uptake downstream of the P2X7R in primary mouse macrophages and human THP-1 cells [65]. Therefore, we sought to determine the role of ANO6 on BzATP-induced YO-PRO-1 uptake in primary human microglia. We discovered that the nonselective Cl^−^ channel blockers tannic acid (20 μM) and DIDS (100 μM) strongly inhibited YO-PRO-1 uptake (Fig. 9a) without affecting the ATP-gated membrane current (Fig. 9b, c) or the ability of this current to facilitate during repetitive applications (Fig. 9d). The fact that tannic acid and DIDS block YO-PRO-1 uptake but not ATP-gated membrane current supports the hypothesis that the P2X7R channel does not form the physical pore through which large cationic dyes enter the cell.

Next, we investigated the role of Ca^2+^ in mediating membrane permeabilization because ATP increases [Ca^2+^]_i_ in human microglia (see Fig. 4) and ANO6 is a Ca^2+^-activated Cl^−^ channel. We incubated microglia in 10 μM BAPTA-AM for 30 min, and then tested the ability of 300 μM BzATP to permeabilize cells bathed in a Ca^2+^-free solution containing 1 mM EDTA. In control experiments using cultured human microglia transiently expressing the genetic Ca^2+^ indicator, GCaMP5, we confirmed that microglia loaded with BAPTA and bathed in EDTA show no change in [Ca^2+^]_i_ in response to BzATP (Additional file 6: Figure S5). Despite this fact, we measured strong YO-PRO-1 fluorescence under these conditions (Fig. 9a). The fact that permeabilization persists in the absence of a change in [Ca^2+^]_i_ argues against a role for ANO6 in the ATP-dependent pathway in cultured human microglia.

### K^+^ efflux triggers Cl^−^ channel-mediated dye uptake

In mouse macrophages, P2X7Rs are thought to trigger dye uptake by depleting intracellular K^+^ [66]. We tested the dependence of dye uptake on K^+^ depletion in cultured human microglia using the K^+^/H^+^ ionophore nigericin. Stimulation with 20 μM nigericin for 30 min resulted in robust uptake of YO-PRO-1 (Fig. 9e). The dye uptake was not due to cell lysis since nigericin did not stimulate LDH release (Additional file 5: Figure S4d). Interestingly, nigericin-stimulated dye uptake was inhibited by tannic acid (Fig. 9f, g), demonstrating that the effect of nigericin occurs upstream of the Cl^−^ channel activation.

## Discussion

In this study, we investigated the ability of the P2X7 receptor to drive permeabilization and activation of cultured adult human microglia. We were motivated by the relative lack of information regarding the effects of ATP on primary human microglia by comparison to conclusions drawn from work on small animal rodent models. We used cultured human microglia proliferating in a nutritive medium containing fetal calf serum and M-CSF as an in vitro model to probe the effects of ATP. Our decision to use a proliferating system arose from the simple fact that it is not easy to obtain a steady source of primary microglia from CNS surgeries. However, it is important to note that our cultured cells lack key components of the purinergic component of the innate immune response; among these are the absence of functional hP2X4Rs in the cell surface membrane and the lack of pro- and mature IL-1β in cell lysates. The presence of the P2X4R is predicted from work on murine microglia [67, 68], where LPS increases functional expression of this receptor in the surface membrane [43]. In contrast, we saw no evidence of ATP-gated P2X4R currents in cultured human microglia before or after LPS treatment. While this may represent a genuine difference between murine and human cells, it is also possible that the P2X4R gene, which may be present and active in human cells in situ [26], is downregulated under the culture conditions in which our cells were maintained. Indeed, significant changes in gene expression occur in both mouse and human microglia upon placing these cells in culture [69–71]. Downregulation could also explain our failure to measure microvesiculation, phosphatidylserine translocation, and the release of IL-1β and IL-18 in response to application of ATP. Thus, we do not draw firm conclusions regarding the differences in phenotype of the actions of ATP on cultured rodent and human microglia. Future experiments using defined, serum-free media to maintain short-term cultures of non-proliferating microglia in an environment that more closely mimics the native milieu may provide a clearer picture of glial pharmacology and physiology although, again, such experiments depend on a reliable source of primary tissue.

Despite these problems, our model presents advantages that facilitate the study of the human phenotype. M-CSF and serum-driven cell proliferation provide a steady source of human microglia that survive multiple splits without measurable changes in the response to ATP. Further, the cells display several of the characteristics expected for microglia in situ including ATP-gated cation current, membrane permeabilization, and inhibition of phagocytosis.

We found that extracellular ATP gates a non-desensitizing inward current at physiological membrane potentials that gradually facilitates with repetitive applications of agonist. The phenotype and pharmacological profile of the response suggest a uniform population of functional P2X7Rs. Further, we find that sustained applications induce membrane permeabilization to polyatomic ions. Although ATP-induced permeabilization is a well-documented phenomenon [72], this report is the first to demonstrate the effect in cultured human microglia. Our main findings on permeabilization are that (i) activation of the P2X7R results in the selective uptake of large molecular weight cationic dyes, (ii) membrane permeabilization is triggered by K^+^ efflux and is independent of a change in [Ca^2+^]_i_, and (iii) permeabilization requires downstream protein(s), one of which may be an unspecified Cl^−^ channel.

The exact mechanism underlying the ability of the P2X7R to stimulate dye uptake in multiple cell types is unresolved [73]. P2X receptors show a small but measurable baseline permeability to large organic cations [74, 75] including YO-PRO-1 [58, 76] which, over the course of a 15–30-min application of ATP, might be large enough to produce the fluorescent changes reported here [77]. However, we found uptake of YO-PRO-1 was blocked by drugs (tannic acid, DIDS) that have no effect on ATP-gated membrane currents, suggesting the involvement of additional pathways. We investigated two such pathways previously reported to facilitate uptake of large cations and anions in mice [55, 56, 78]. The first was pannexin-1, which is thought to be responsible for permeabilization of rodent neurons and astrocytes [64, 79], and the second is the Ca^2+^-activated Cl^−^ channel, ANO6, which is active in mouse and human macrophages [65]. Protocols designed to block these channels (pannexin antagonists and Ca^2+^ chelation, respectively) had no effect on YO-PRO-1 uptake in our experiments, providing practical evidence that they play no role in permeabilization of cultured human microglia. However, it is possible that ATP activates ANO6 independent of a change in [Ca^2+^]_i_. ATP and P2X7Rs activate a Ca^2+^-independent phospholipase A2 in murine macrophages [80], an enzyme capable of activating ANO6 in heterologous expression systems [81]. Future experiments designed to explore this possibility should be pursued.

We found two non-specific Cl^−^ channel blockers (tannic acid and DIDS) that inhibited YO-PRO-1 uptake triggered by BzATP and nigericin without affecting currents carried by small cations. We draw two important conclusions from these experiments. First, while it is probable that YO-PRO-1 permeates the P2X7R [58], it is unlikely that this channel forms the dominate pathway for the flow of large cations into cultured human microglia under the conditions of the experiments presented here. Second, it is possible that an undefined Cl^−^ channel plays a role in the signaling cascade. Because tannic acid blocks the YO-PRO-1 uptake triggered by nigericin, we suggest that this Cl^−^ channel sits downstream of the P2X7R. Therefore, we propose a pathway where efflux of K^+^ from the P2X7R serves to activate a downstream protein, which may be a Cl^−^ channel, that is required for microglial uptake of large organic cations.

The gene expression patterns we measured from microglia cultured in serum suggest a phagocytotic phenotype, a result in keeping with the idea that serum exposure promotes phagocytosis in cultured rat microglia [69]. In fact, we measured robust phagocytosis of *E. coli* particles that was inhibited by extracellular ATP. In human monocytes and monocyte-derived macrophages, P2X7Rs serve as scavenger receptors that aid in engulfment of bacteria and apoptotic cells via intercellular thiol-disulfide exchange reactions, the extracellular domain of the P2X7R, and newly exposed epitopes on the apoptotic target [21, 46, 82]. Upon attachment of apoptotic target cells to the P2X7R , associated non-muscle mysosin IIA triggers rearrangement of the cytoskeleton and engulfment of the target [47]. In the presence of ATP, non-muscle myosin dissociates from the P2X7R and phagocytosis is decreased [48]. Our work to date showing inhibition of phagocytosis by ATP suggests that a similar mechanism is active in cultured human microglia. Therefore, future experiments designed to study the role of the P2X7R as a scavenger receptor in these cells are warranted, including work that examines whether P2X7R-mediated inhibition of phagocytosis proceeds through myosin dissociation.

Finally, we found that P2X7Rs of cultured human microglia are unable to stimulate the release of IL-1β or IL-18 pro-inflammatory cytokines. The lack of cytokine release is in keeping with the proposed anti-inflammatory phenotype of the cultured cells. Previous work established that the Glu^496^ to Ala polymorphism in the P2X7R causes significant reduction in ATP-evoked ethidium uptake and IL-1β release from human monocytes of homozygous donors [49, 83]. In the present study, microglia from donors heterozygous at ^496^Glu/Ala showed significant ATP-gated membrane current and robust uptake of YO-PRO-1, demonstrating P2X7R functionality. Thus, we propose that the polymorphism does not explain our inability to detect expression of pro-IL-1β after priming with LPS or *E. coli* particles or why treatment of LPS-primed human microglia with nigericin fails to induce IL-1β production and release. Instead, we favor the hypothesis that human microglial cells cultured in serum adopt an “M2-like” state that favors phagocytosis and prevents transcription of pro-IL-1β in response to TLR4 agonists such as LPS. In support of this, we are unable to detect TLR4 expression in the human microglial cells. In light of this data, it is interesting to note that we see substantial production of IL-18. TLR4 stimulation may be nonessential for IL-18 production, as it is constitutively expressed in several cell types [84, 85]. The reason behind the inability of activated caspase-1 to trigger the release of IL-18 is unknown but may result from downregulation of as yet unidentified protein(s). One candidate is gasdermin D, the protein capable of triggering release of both IL-1β and IL-18, as recent reports found gasdermins are expressed in a status-specific manner [86].

The functional consequences of dye permeation are well established in murine microglia. Notably, recent findings indicate that P2X7Rs are potential targets for limiting neuroinflammation [87]. Further, P2X7R stimulation of Cl^−^ channels and consequent dye uptake in murine macrophages enhances phagocytosis and bacterial killing, stimulates membrane blebbing and phospholid scrambling, and induces delayed apoptosis [65]. Thus, our data warrants further investigation into the functional consequences of human microglia permeabilization, which may also impact studies of other types of human cells that express the P2X7R including human macrophages, mastocytes, dendritic cells, astrocytes, and neurons.

## Conclusions

The present results provide evidence that the P2X7R displays dual roles as both a cationic channel and mediator of cellular permeabilization in cultured primary human microglia. The study was motivated by a wealth of previous literature demonstrating that the ability of the P2X7R to permeabilize murine microglia is essential for NLRP3 inflammasome activation and subsequent IL-1β release, microglia proliferation, and production of reactive oxygen species [18, 25]. Our results indicate that the P2X7R also mediates innate immunity in human microglia. Specifically, P2X7Rs reduced microglial phagocytic capacity and produced mature caspase-1 by activating the inflammasome. Consequently, the P2X7R is likely to promote inflammation in the human CNS. We also found that the P2X7R has the unique ability to permeabilize human microglia cells which allows the uptake of large molecular weight dyes. Our data indicate that this uptake pathway is selective for cations such as YO-PRO-1 and requires an unspecified Cl^−^ channel. Activation of the Cl^−^ channel responsible for dye uptake is dependent on K^+^ efflux and independent of Ca^2+^ flux. Taken together, we demonstrate that the P2X7R contributes to a diverse array of human microglial functions. The fact that P2X7Rs subserve multiple pathophysiological functions in human microglia suggests that antagonists of this receptor are potential therapeutic reagents for disorders of neuroinflammation.

## Additional files

Additional file 1: **Figure S1.** Ivermectin potentiates P2X7R current. ATP was applied every 15 s in the absence and presence of 20 μM ivermectin. (a) 100 μM ATP (green bars) failed to evoke significant membrane current even in the presence of ivermectin (orange bar). In contrast, 10 mM ATP (light blue bars) evoked robust inward currents. (b) The current evoked by 10 mM ATP is potentiated by co-application of 20 μM ivermectin. (EPS 5969 kb)

Additional file 2: **Figure S2.** Microglia P2X7Rs are dynamically regulated by bacterial phagocytosis. (a) The current-voltage (*I*-*V*) relation from control and *E. coli*-positive human microglia was obtained by 500 ms ramp pulses from a holding potential of − 60 mV, initially hyperpolarized to − 90 mV, then depolarized to + 30 mV, and hyperpolarized back to the holding potential. (b) Representative tracing of current rundown recorded from a phagocytic microglia cell stimulated with successive applications of 10 mM ATP (3 s duration) separated by 15-s intervals, from a holding potential of − 60 mV. (EPS 4895 kb)

Additional file 3: **Figure S3.** P2X7Rs do not regulate microglial intracellular pH, IL-1β/IL-18 release, blebbing, or phosphatidylserine switch. Human microglia were treated with or without BzATP (300 μM) overnight, and intracellular pH was measured by staining with pHrodo Green AM Intracellular pH Indicator (30 min, 37 °C). (a) Quantification of intracellular pH RFU reveals there was no significant difference in RFU between control and BzATP-treated cells, *n* = 4 separate experiments. (b) THP-1 monocytes were treated with 1 μg/mL LPS for 4 h followed by BzATP (300 μM, 30 min) or nigericin (20 μM, 3 h) treatment. The supernatant was collected and analyzed for IL-1β by ELISA (*n* = 2 separate experiments). (c) Primary human microglia and HEK293 cells transiently expressing hP2X7R were treated with 500 μM BzATP for 15 min, and the percentage of blebbing cells was quantified (*n* = 4 separate experiments). (d) Primary human microglia were incubated with or without *E. coli* particles ± BzATP (300 μM) and stained for annexin V. The percentage of annexin V cells was quantified, and there was no significant difference between groups (*n* = 4 separate experiments). (e) Human microglia were treated with 10 μg/mL LPS for 3 or 24 h followed by BzATP (300 μM, 30 min) or nigericin (20 μM, 3 h) treatment. The cell supernatants and lysates were collected and analyzed for IL-18 by ELISA (*n* = 2 separate experiments). The graph represents cell lysate IL-18 levels; no IL-18 was detected in cell supernatants. (EPS 5031 kb)

Additional file 4: Cultured human microglia do not bleb in response to ATP. Microglia cultured on chamber slides were exposed to 300 μM BzATP. Confocal microscopy was used to capture image morphology at 15-s intervals for 30 min after BzATP application. Scale bar = 20 μm. (GIF 1351 kb)

Additional file 5: **Figure S4.** P2X7R dye uptake is independent of cell lysis and selective for cations below a certain threshold of molecular weight. (a) Time course of ethidium uptake shows the average s.e.m. of fluorescence intensity over time measured from individual cells within a single field of view after application of 300 μM BzATP (red trace) compared to unstimulated microglia (black trace). (b) Quantification of ethidium uptake by microglia reveals significant stimulation of dye uptake by BzATP (300 μM) for 25 separate experiments. Significance determined using an unpaired *t* test. (c) Quantitative comparison of YOYO-1 uptake by microglia. Cells were incubated for 15 min with YOYO-1 and 300 μM BzATP. There was no significant uptake of the dye, *n* = 4 separate experiments. As a positive control, microglia were lysed with 0.5% Triton X-100 and displayed robust uptake of YOYO-1. (d) Human microglia were treated with BzATP (300 μM), ATP (5 mM), or nigericin (20 μM) for 30 min at 37 °C. The negative control was obtained from untreated microglia, and the positive control was from lysed microglia. After treatment, cell supernatants were collected and LDH release was quantified. Percent cytotoxicity is the amount of LDH release normalized to the positive control; there was no significant difference between the negative control and BzATP, ATP, or nigericin-treated microglia (*n* = 3 separate experiments). (EPS 5922 kb)

Additional file 6: **Figure S5.** BAPTA-AM prevents changes in [Ca^2+^]_i_. Primary human microglia were transfected with GCaMP5 using the Effectene Transfection Reagents (4 μl cDNA, 8 μl enhancer, 16 μl effectene) and used 48 h later for [Ca^2+^]_i_ measurements. Microglia were incubated in ECS containing 10 μM BAPTA-AM and 2 μl pluronic acid for 60 min at 37 °C, and then left to rest for 30 min. GCaMP5 fluorescence was measured using a GFP filter set. (a) Primary human microglia show a low level of fluorescence at rest, indicating a low resting [Ca^2+^]_i_. (b) Adding 300 μM BzATP to the ECS increases fluorescence as [Ca^2+^]_i_ rises. (c) Cells incubated in BAPTA-AM show a low level of resting [Ca^2+^]_i_. (d) Addition of BzATP to the cell shown in panel C causes no change in [Ca^2+^]_i_. (EPS 36386 kb)

## Abbreviations

ATP: Adenosine triphosphate
CBX: Carbenoxolone
DAMP: Damage-associated molecular pattern
ECS: Extracellular solution
*I*_ATP_: ATP-gated current
P2XR: P2X receptor
RFU: Relative fluorescence unit
TA: Tannic acid
