# Supplementary figures and images for: A central role for P2X7 receptors in human microglia
Source: J Neuroinflammation. 2018 Nov 21;15:325. doi: 10.1186/s12974-018-1353-8 (PMC6247771; doi:10.1186/s12974-018-1353-8)

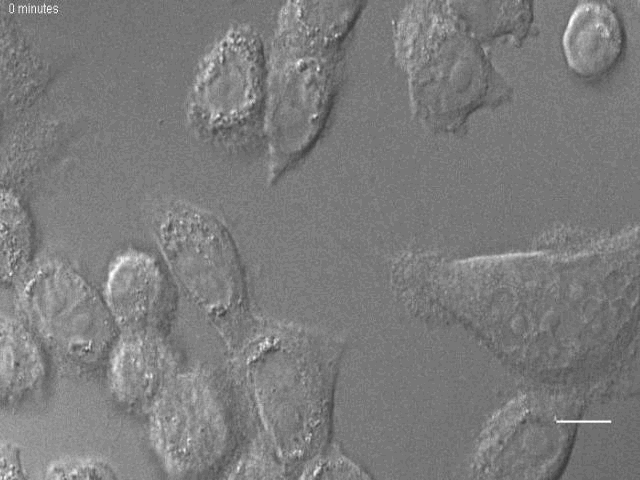

Supplement: Supplementary file 4 — Cultured human microglia do not bleb in response to ATP. Microglia cultured on chamber slides were exposed to 300 μM BzATP. Confocal microscopy was used to capture image morphology at 15-s intervals for 30 min after BzATP application. Scale bar = 20 μm. (GIF 1351 kb) [file 12974_2018_1353_MOESM4_ESM.gif]
